# Supplementary material for: The SWI/SNF chromatin remodeling assemblies BAF and PBAF differentially regulate cell cycle exit and cellular invasion in vivo
Source: PLoS Genet. 2022 Jan 4;18(1):e1009981. doi: 10.1371/journal.pgen.1009981 (PMC8759636; doi:10.1371/journal.pgen.1009981)
Supplement: S1 Fig — Single planes of confocal z-stacks representing AC invasion in swsn-1(os22) and swsn-4(os13) temperature sensitive mutants with fluorescently labeled AC (magenta, cdh-3>mCherry::moeABD) and BM (green, laminin::GFP) scored at the permissive temperature (A) and restrictive temperature (B). Significant loss of invasion was seen in both swsn-1(os22) (20% loss of invasion) and swsn-4(os13) (24% loss of invasion) hypomorphicts strains when grown at the restrictive temperature 25°C and assessed at the P6.p 4-cell 1° VPC stage (B). White arrowheads indicate ACs. Yellow arrowheads in A indicate boundaries of breaches in the BM. Numbers in bottom right of fluorescence overlay panel in A indicate penetrance of wildtype AC invasion. Numbers in bottom right of fluorescence overlay panel in B indicate penetrance of invasion defects. (PDF) [file pgen.1009981.s001.pdf]

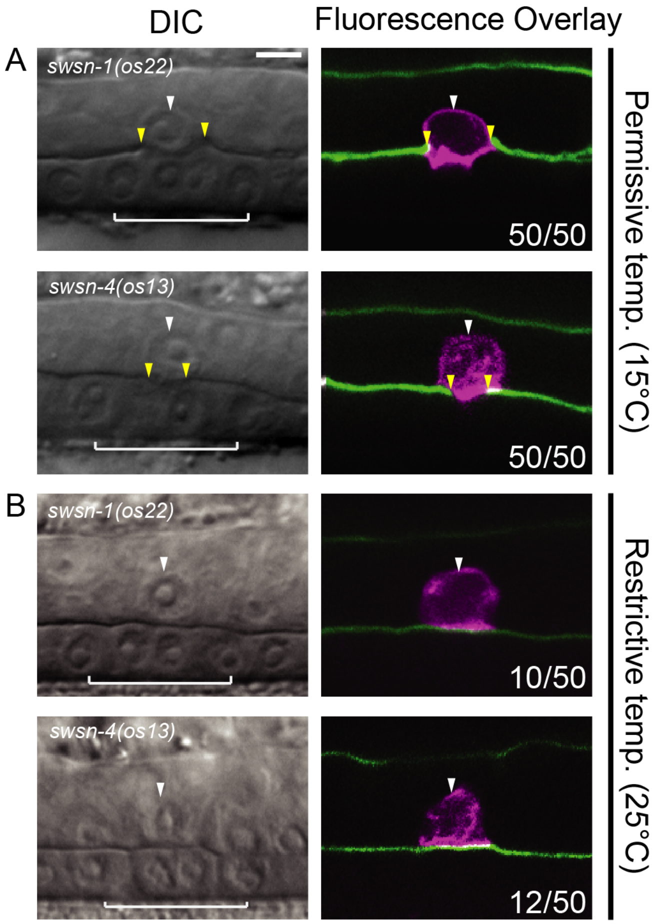

Figure S1. AC invasion is disrupted in temperature sensitive SWI/SNF hypomorphs. Single planes of confocal z-stacks representing AC invasion in *swsn-1(os22) and swsn-4(os13)* temperature sensitive mutants with fluorescently labeled AC (magenta, *cdh-3>mCherry::moeABD*) and BM (green, *laminin::GFP*) scored at the permissive temperature **(A)** and restrictive temperature **(B)**. Significant loss of invasion was seen in both *swsn-1(os22)* (20% loss of invasion) and *swsn-4(os13)* (24% loss of invasion) hypomorphic^{ts} strains when grown at the restrictive temperature 25°C and assessed at the P6.p 4-cell 1° VPC stage **(B)**. White arrowheads indicate ACs. Yellow arrowheads in A indicate boundaries of breaches in the BM. Numbers in bottom right of fluorescence overlay panel in A indicate penetrance of wildtype AC invasion. Numbers in bottom right of fluorescence overlay panel in B indicate penetrance of invasion defects.
