# Supplementary material for: The SWI/SNF chromatin remodeling assemblies BAF and PBAF differentially regulate cell cycle exit and cellular invasion in vivo
Source: PLoS Genet. 2022 Jan 4;18(1):e1009981. doi: 10.1371/journal.pgen.1009981 (PMC8759636; doi:10.1371/journal.pgen.1009981)
Supplement: S2 Fig — Fluorescent micrographs depicting BM (laminin::GFP) and expression of SWSN-4::GFP (A), SWSN-8::GFP (B), and PBRM-1::eGFP (C) in the AC in animals fed empty vector control (left) or RNAi targeting the endogenous allele (right). White arrowheads indicate ACs, yellow arrowheads indicate boundaries of breach in BM, and white brackets indicate 1 VPCs. Scale bar, 5μm. (D) Corresponding quantifications of fluorescent expression. Statistical comparisons were made between the expression of each SWI/SNF subunit in the AC in control and RNAi-treated animals using Student’s t-test (n≥30 for each stage and subunit; p values are displayed above compared data). (E) Stacked bar chart showing percentage of AC invasion defects corresponding to each treatment, binned by AC phenotype (n≥30 animals per condition). (PDF) [file pgen.1009981.s002.pdf]

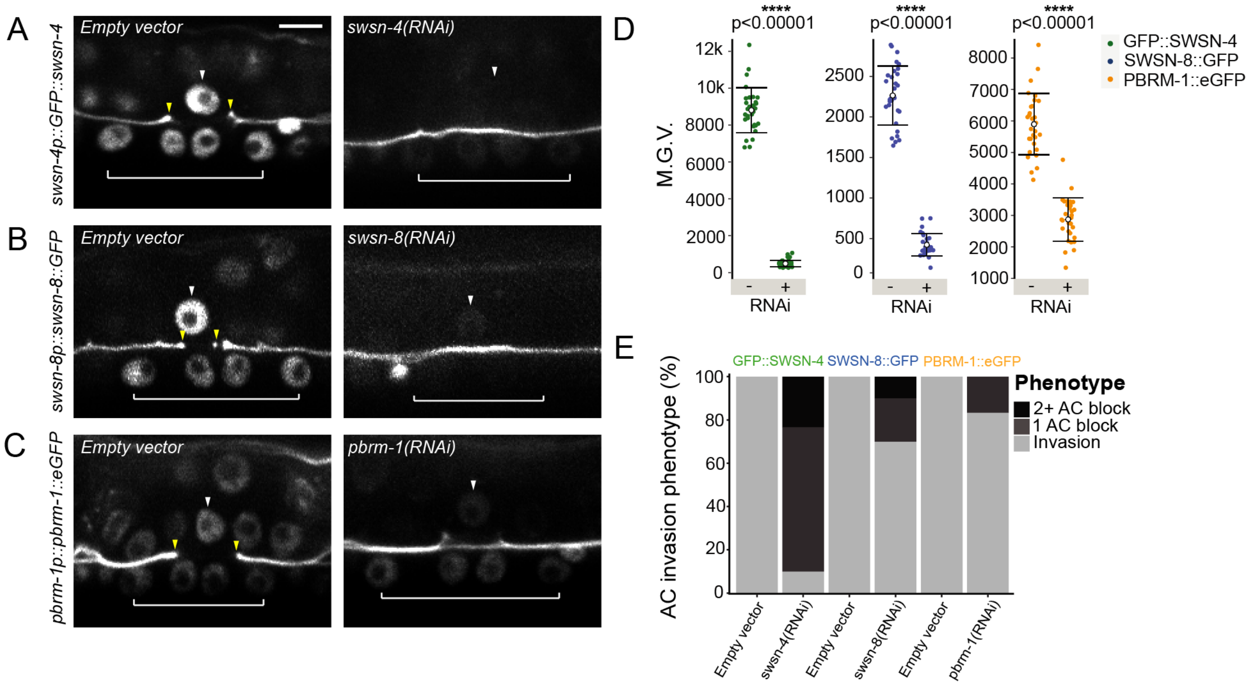

Figure S2. Improved SWI/SNF RNAi significantly knocks down SWI/SNF expression in the AC. Fluorescent micrographs depicting BM (*laminin::GFP*) and expression of SWSN-4::GFP **(A)**, SWSN-8::GFP **(B)**, and PBRM-1::eGFP **(C)** in the AC in animals fed empty vector control (left) or RNAi targeting the endogenous allele (right). White arrowheads indicate ACs, yellow arrowheads indicate boundaries of breach in BM, and white brackets indicate 1 VPCs. Scale bar, 5µm. **(D)** Corresponding quantifications of fluorescent expression. Statistical comparisons were made between the expression of each SWI/SNF subunit in the AC in control and RNAi-treated animals using Student's *t*-test (n≥30 for each stage and subunit; p values are displayed above compared data). **(E)** Stacked bar chart showing percentage of AC invasion defects corresponding to each treatment, binned by AC phenotype (n≥30 animals per condition).
