# Supplementary material for: The SWI/SNF chromatin remodeling assemblies BAF and PBAF differentially regulate cell cycle exit and cellular invasion in vivo
Source: PLoS Genet. 2022 Jan 4;18(1):e1009981. doi: 10.1371/journal.pgen.1009981 (PMC8759636; doi:10.1371/journal.pgen.1009981)
Supplement: S3 Fig — (A-C) Representative fluorescence micrographs depicting endogenous GFP expression of individual SWI/SNF subunits representative of the core (swsn-4, A), BAF assembly (swsn-8, B), and PBAF assembly (pbrm-1, C) in the AC (cdh-3p::mCherry::moeABD) following treatment with RNAi targeting each SWI/SNF assembly (A), or the core ATPase and alternative SWI/SNF assembly (B-C). White arrowheads indicate ACs, yellow arrowheads indicate boundaries of breach in BM, and white brackets indicate 1 VPCs. Scale bar, 5μm. (D) Quantification of fluorescence expression (mean gray value) of endogenous subunits in each condition. Data is normalized to empty vector control across each strain. Statistical comparisons were made between the expression of each SWI/SNF subunit in the AC in control and RNAi-treated animals using Student’s t-test (n≥30 for each stage and subunit; p values are displayed above compared data). n.s. not significant. (E) Quantification of fluorescence expression of endogenous GFP-tagged subunits of non-invasive ACs following loss of expression of alternative SWI/SNF subunits, binned per RNAi treatment by phenotype into single non-invasive AC (1AC) and mitotic non-invasive AC (2+ AC). Statistical comparisons (Student’s t-test; p values are displayed above compared data) were limited to conditions with n>10 ACs in each phenotype. n.s. not significant. (F) Schematic summary of SWI/SNF core and assembly auto and cross regulation. (PDF) [file pgen.1009981.s003.pdf]

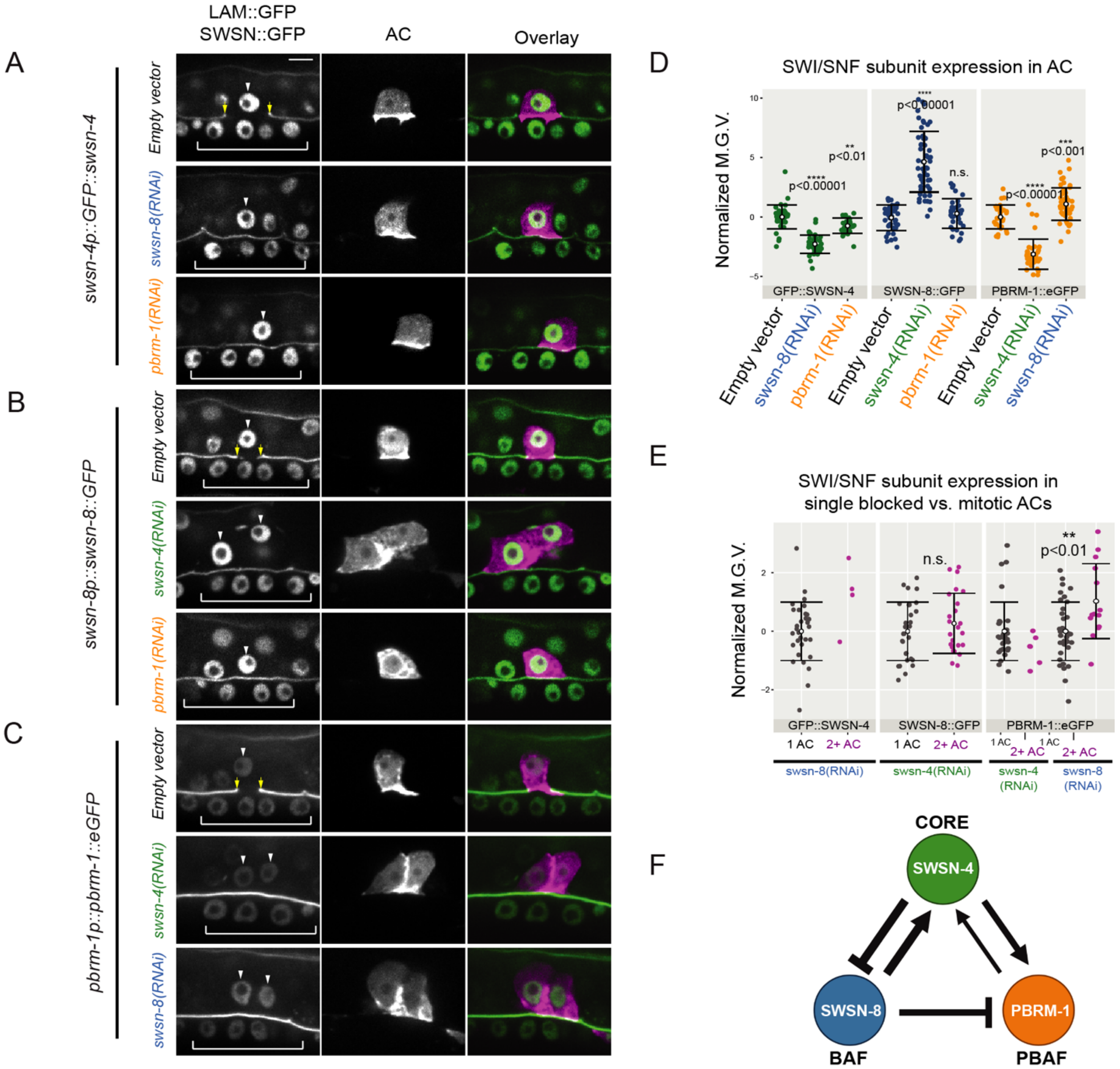

Figure S3. SWI/SNF subunits exhibit intra-complex and inter-assembly regulation. (A-C) Representative fluorescence micrographs depicting endogenous GFP expression of individual SWI/SNF subunits representative of the core (*swsn-4*, **A**), BAF assembly (*swsn-8,* **B**), and PBAF assembly (*pbrm-1*, **C**) in the AC (*cdh-3p::mCherry::*moeABD) following treatment with RNAi targeting each SWI/SNF assembly **(A),** or the core ATPase and alternative SWI/SNF assembly **(B-C)**. White arrowheads indicate ACs, yellow arrowheads indicate boundaries of breach in BM, and white brackets indicate 1 VPCs. Scale bar, 5µm. **(D)** Quantification of fluorescence expression (mean gray value) of endogenous subunits in each condition. Data is normalized to empty vector control across

each strain. Statistical comparisons were made between the expression of each SWI/SNF subunit in the AC in control and RNAi-treated animals using Student's *t*-test ($n \geq 30$ for each stage and subunit; p values are displayed above compared data). n.s. not significant**. (E)** Quantification of fluorescence expression of endogenous GFP-tagged subunits of non-invasive ACs following loss of expression of alternative SWI/SNF subunits, binned per RNAi treatment by phenotype into single non-invasive AC (1AC) and mitotic non-invasive AC (2+ AC). Statistical comparisons (Student's *t*-test; p values are displayed above compared data) were limited to conditions with $n > 10$ ACs in each phenotype. n.s. not significant. **(F)** Schematic summary of SWI/SNF core and assembly auto and cross regulation.
