# Supplementary material for: The SWI/SNF chromatin remodeling assemblies BAF and PBAF differentially regulate cell cycle exit and cellular invasion in vivo
Source: PLoS Genet. 2022 Jan 4;18(1):e1009981. doi: 10.1371/journal.pgen.1009981 (PMC8759636; doi:10.1371/journal.pgen.1009981)
Supplement: S4 Fig — (A) Single confocal z-planes depicting DIC (left) and expression of lineage-restricted CDK sensor (unc-62>DHB::2xmKate2, right) in the vulva and SM cells at the P6.p 8-cell stage corresponding to the stage when wild-type SM cells differentiate and exit the cell cycle. Animals were treated with empty vector control (top) or swsn-4(RNAi) (bottom). All representative images in each treatment are derived from the same z-stack from the same animal in the corresponding z-plane (top-left). Average or individual C/N CDK sensor ratios are listed in the bottom-right of corresponding panels. White arrowheads indicate individual SM cells. White brackets indicate 1° VPCs. (B) Quantification of the number of SM cells present at the P6.p 8-cell stage in control and swsn-4(RNAi) treated animals. (C) C/N CDK sensor ratios for SM cells in each treatment. Gradient scale depicts cell cycle state as determined by quantification of each AC in all treatments (n≥30 animals per treatment), with dark-black depicting differentiation into G0/G1 and lighter-magenta depicting G2 cell cycle states. (PDF) [file pgen.1009981.s004.pdf]

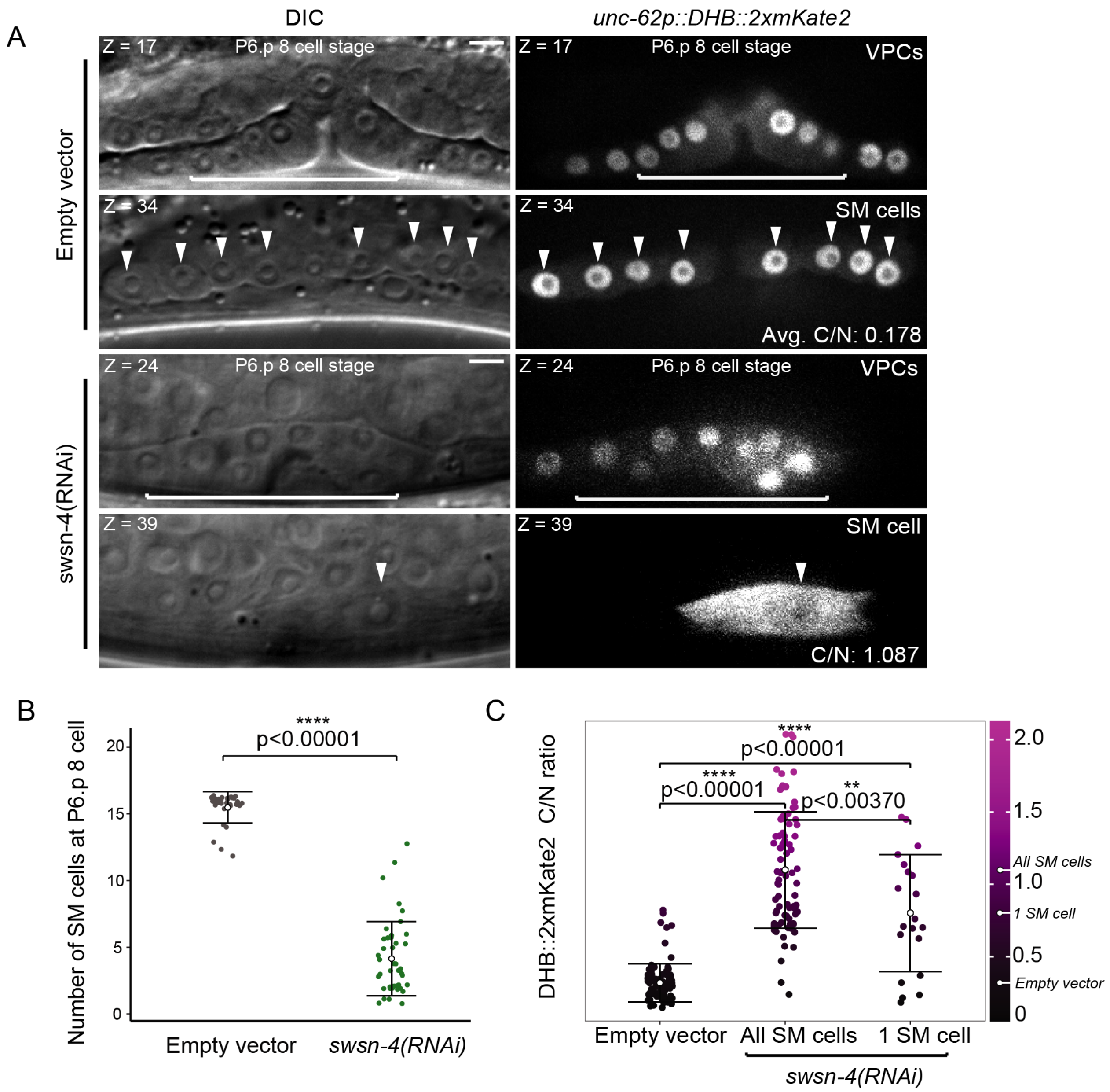

Figure S4. Improved *swsn-4* RNAi recapitulates SWI/SNF ATPase null phenotype in the sex myoblasts. (A) Single confocal z-planes depicting DIC (left) and expression of lineage-restricted CDK sensor (*unc-62>DHB::2xmKate2*, right) in the vulva and SM cells at the P6.p 8-cell stage corresponding to the stage when wild-type SM cells differentiate and exit the cell cycle. Animals were treated with empty vector control (top) or *swsn-4(RNAi)* (bottom). All representative images in each treatment are derived from the same z-stack from the same animal in the corresponding z-plane (top-left). Average or individual C/N CDK sensor ratios are listed in the bottom-right of corresponding panels.

White arrowheads indicate individual SM cells. White brackets indicate 1° VPCs. **(B)** Quantification of the number of SM cells present at the P6.p 8-cell stage in control and *swsn-4(RNAi)* treated animals. **(C)** C/N CDK sensor ratios for SM cells in each treatment. Gradient scale depicts cell cycle state as determined by quantification of each AC in all treatments (n≥30 animals per treatment), with dark-black depicting differentiation into G_0/G_1 and lighter-magenta depicting G_2 cell cycle states.
