# Supplementary material for: The SWI/SNF chromatin remodeling assemblies BAF and PBAF differentially regulate cell cycle exit and cellular invasion in vivo
Source: PLoS Genet. 2022 Jan 4;18(1):e1009981. doi: 10.1371/journal.pgen.1009981 (PMC8759636; doi:10.1371/journal.pgen.1009981)
Supplement: S5 Fig — (A) DIC (left) and fluorescent (right) images depicting BM (laminin::GFP) and AC-specific CKI-1 (cdh-3>CKI-1::GFP) in empty vector control animal (top) and animals treated with swsn-4(RNAi) (middle) or swsn-8(RNAi) (bottom). Scale bar, 5μm. (B) Stacked bar chart showing quantification of percentage of AC invasion defects corresponding to each treatment (n≥30 animals per condition, p values for Fisher’s exact test comparing invasion penetrance in control animals and animals with the rescue transgene (+CKI-1::GFP) are displayed above black brackets). (PDF) [file pgen.1009981.s005.pdf]

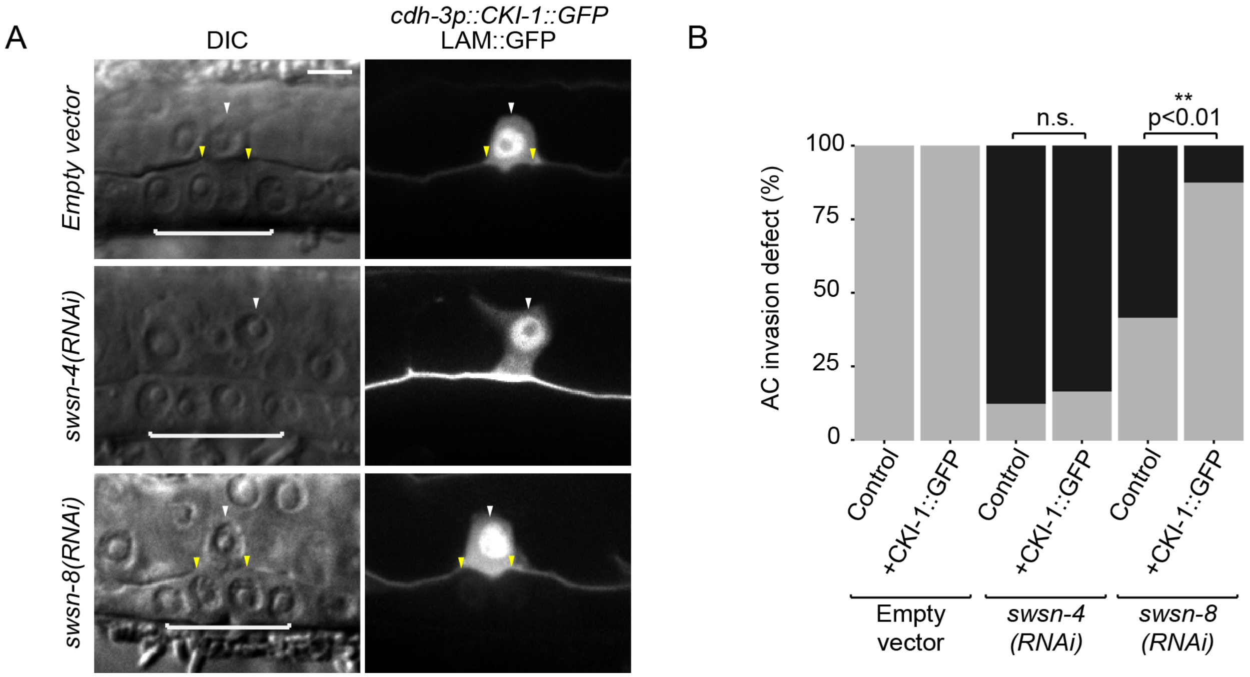

Figure S5. AC-specific expression of CKI-1 rescues invasion in BAF-depleted ACs. **(A)** DIC (left) and fluorescent (right) images depicting BM (*laminin::GFP*) and AC-specific CKI-1 (*cdh-3>CKI-1::GFP*) in empty vector control animal (top) and animals treated with *swsn-4(RNAi)* (middle) or *swsn-8(RNAi)* (bottom). Scale bar, 5µm. **(B)** Stacked bar chart showing quantification of percentage of AC invasion defects corresponding to each treatment (n≥30 animals per condition, p values for Fisher's exact test comparing invasion penetrance in control animals and animals with the rescue transgene (+CKI-1::GFP) are displayed above black brackets).
