# Supplementary material for: The SWI/SNF chromatin remodeling assemblies BAF and PBAF differentially regulate cell cycle exit and cellular invasion in vivo
Source: PLoS Genet. 2022 Jan 4;18(1):e1009981. doi: 10.1371/journal.pgen.1009981 (PMC8759636; doi:10.1371/journal.pgen.1009981)
Supplement: S6 Fig — (A) Representative DIC (left) and fluorescent (right) micrographs depicting expression of endogenous GFP::FOS-1a and BM (laminin::GFP) in control (top) and pbrm-1(RNAi) treated (bottom) animals. White arrowhead indicates ACs, yellow arrowheads indicate boundaries of the breach in the BM. Scale bar, 5μm. (B) Quantification of GFP::FOS-1a expression in ACs of control and pbrm-1(RNAi) treated animals, normalized to mean expression of control group. Statistical comparisons were made between expression in the AC in control and RNAi-treated animals using Student’s t-test (n≥20 for each condition; p value is displayed above black bracket). (C) DIC-Fluorescence overlay (left), and PBRM-1::mNG::AID and BM (LAM::GFP) (right), in animals treated with empty vector control (top) or fos-1(RNAi) (bottom). (D) Stacked bar chart showing percentage of AC invasion defects corresponding to each treatment and genetic background in C (n≥30 animals per condition, p values for Fisher’s exact test comparing invasion defect penetrance in wild-type animals treated with fos-1(RNAi) and pbrm-1::mNG::AID animals treated with fos-1(RNAi) is displayed above black bracket). (E) Representative DIC (top-left), BM (LAM::GFP, top-right), AC (cdh-3>PH, bottom-left), and overlay (bottom-right) of P6.p 8-cell vulva in an MMP-deficient (-) animal treated with pbrm-1(RNAi). (F) Stacked bar chart showing percentage of AC invasion defects corresponding to each treatment and genetic background in E (n≥30 animals per condition, p values for Fisher’s exact test comparing invasion defect penetrance in wild-type animals treated with pbrm-1(RNAi) and MMP(-) animals treated with pbrm-1(RNAi) is displayed above black bracket). (PDF) [file pgen.1009981.s006.pdf]

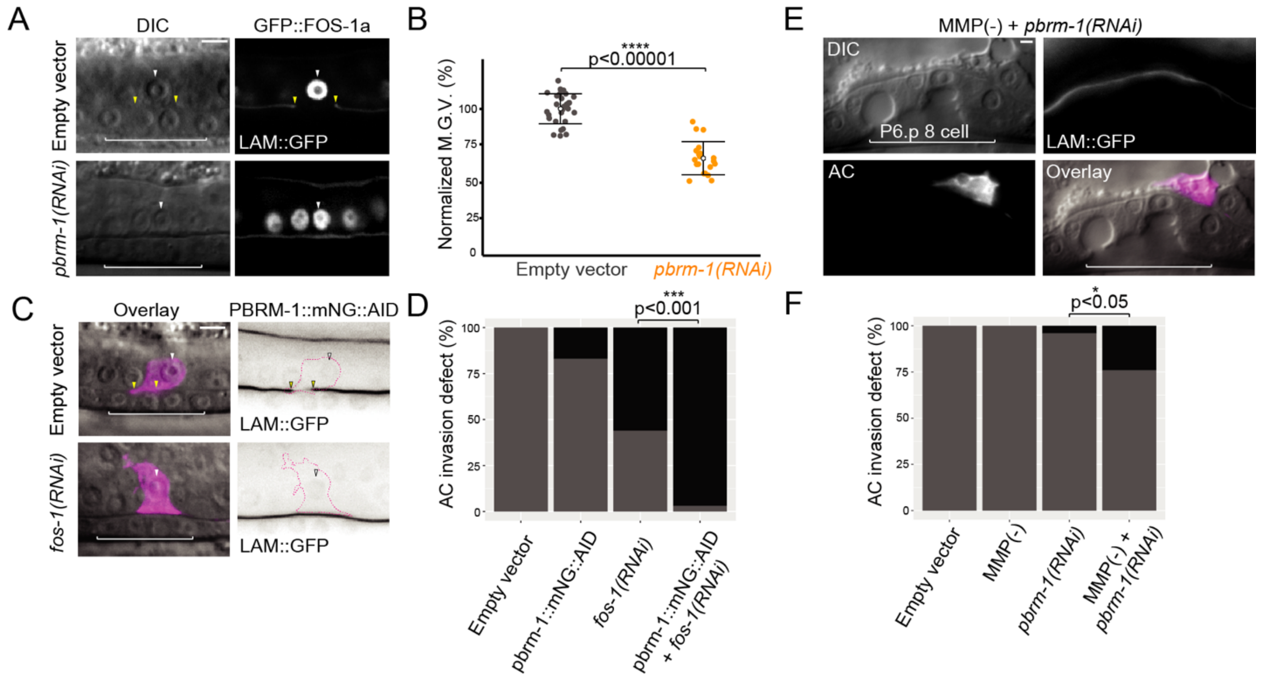

Figure S6. PBAF partially regulates the FOS-1 transcription factor. (A) Representative DIC (left) and fluorescent (right) micrographs depicting expression of endogenous GFP::FOS-1a and BM (*laminin::GFP*) in control (top) and *pbrm-1(RNAi)* treated (bottom) animals. White arrowhead indicates ACs, yellow arrowheads indicate boundaries of the breach in the BM. Scale bar, 5µm. **(B)** Quantification of GFP::FOS-1a expression in ACs of control and *pbrm-1(RNAi)* treated animals, normalized to mean expression of control group. Statistical comparisons were made between expression in the AC in control and RNAi-treated animals using Student's *t*-test (n≥20 for each condition; p value is displayed above black bracket). **(C)** DIC-Fluorescence overlay (left), and PBRM-1::mNG::AID and BM (LAM::GFP) (right), in animals treated with empty vector control (top) or *fos-1(RNAi)* (bottom). **(D)** Stacked bar chart showing percentage of AC invasion defects corresponding to each treatment and genetic background in C (n≥30 animals per condition, p values for Fisher's exact test comparing invasion defect penetrance in wild-type animals treated with *fos-1(RNAi)* and *pbrm-1::mNG::AID* animals treated with *fos-1(RNAi)* is displayed above black bracket). **(E)** Representative DIC (top-left), BM (LAM::GFP, top-right), AC (*cdh-3>PH*, bottom-left), and overlay (bottom-right) of P6.p 8-cell vulva in an MMP-deficient (-) animal treated with *pbrm-1(RNAi)*. **(F)** Stacked bar chart showing percentage of AC invasion defects corresponding to each treatment

and genetic background in E (n≥30 animals per condition, p values for Fisher's exact test comparing invasion defect penetrance in wild-type animals treated with *pbrm-1(RNAi)* and MMP(-) animals treated with *pbrm-1(RNAi)* is displayed above black bracket).
