# Supplementary material for: The SWI/SNF chromatin remodeling assemblies BAF and PBAF differentially regulate cell cycle exit and cellular invasion in vivo
Source: PLoS Genet. 2022 Jan 4;18(1):e1009981. doi: 10.1371/journal.pgen.1009981 (PMC8759636; doi:10.1371/journal.pgen.1009981)
Supplement: Attachment — Submitted filename: Smith et al 2021 Rebuttal PLoS Genetics.pdf [file pgen.1009981.s014.pdf]

We would like to thank the three anonymous reviewers for providing a critique of our manuscript, "The SWI/SNF chromatin remodeling assemblies BAF and PBAF differentially regulate cell cycle exit and cellular invasion *in vivo."* We are grateful for the thoughtful comments by all three reviewers, which by incorporating their suggestions we hope has made for a stronger manuscript. Please see below the specific responses to individual queries and the track changes version of the manuscript associated with our resubmission to *PLoS Genetics*.

"Reviewer #1: *This is a very interesting study describing the role of SWI/SNF complex in regulating cell cycle exit and invasiveness in the AC cell in C. elegans. The authors use many cutting edge techniques to tackle this question at the level of a single cell in a developing organisms. They show dose dependence of the effect and find differences between the roles of BAP and PBAF. I agree with the initial assessment that identifying the direct targets of BAF and PBAF would add a lot to the story, but I also agree with the authors that that it would not be feasible with currently available techniques. I also think that the experiments as presented advance the field sufficiently to warrant publication in PLoS Genetics. The authors already addressed a set of comments from previous reviewers, and added a fair amount of new data, which did improve the quality of manuscript.* ***I have no major concerns****."*

Minor Comments:

1. **"*Maybe I missed it, but I could not find the in-text citation for the data regarding the role of SWI/SNF in myogenesis in C. elegans, page 6 line 105-106 and page 7 line 107-111. Is there a reference missing? Or did I miss it?"***

   We thank the reviewer for pointing this out. We have updated the manuscript to include the appropriate in-text citation.

"*Reviewer #2:* *This is an interesting (and quite substantial) study of the importance for the SWI/SNF complex in anchor cell invasion, a well-studied model for cell invasion across a basement membrane. It applies a number of cutting edge approaches to this problem including the use of inducible degrons to test for cell-autonomous effects, and the development of CRISPR knock-in alleles to assess gene expression. The authors should be commended for using their GFP CRISPR lines to measure the extent of RNAi-mediated knockdown, something that is unfortunately rarely done in C. elegans studies.*

At a high level, the question of how we can understand the role of pleiotropic regulators like SWI/SNF in specific cells is an important one. While many of the results seem fairly "expected" from prior knowledge, this study does provide new advances. For example, the finding that genes that are part of specific SWI/SNF complexes BAF and PBAF give rise to apparently distinct phenotypes. The endogenously CRISPR-tagged alleles generated here will likely be a useful resource for other C. elegans labs.

The previous reviews for PLOS Biology raised several concerns, that in my view have been adequately addressed for publication in PLOS Genetics."

Major comments:

1. ***"I have some concerns about how specific the results are to SWI/SNF and suggest that this be emphasized a bit more in the framing and interpretation of the paper. The RNAi screen produced a whopping 82 hits out of 269 "chromatin regulatory factors" screened. To me this suggests that almost any major global deficit in gene expression in the anchor cell is likely to lead to defects in invasion. Similarly, it seems likely, especially given the sex mesoblast results, that many/most other cells that undergo dynamic differentiation behaviors would be SWI/SNF dependent. I am curious what blocking RNA PolII e.g. with ama-1 RNAi would have in the anchor cell as sort of an "edge case." Similarly, it is worth posing the question of whether similar SWI/SNF perturbations might similarly block the normal behaviors of other cells that are dynamic after embryogenesis such as Q cell migration, any dividing cells, Y to PDA, FLP/PVD arborization, vulval morphogenesis, etc. I'm hesitant to recommend doing new experiments to ask these questions as the paper already is bending under the weight of a huge amount of data, but they are worth discussing regardless. Obviously this discussion should still account for the fact that hits include both genes predicted to have activating and repressive functions, and the evidence that loss of BAF and PBAF component separately leads to defects in invasion in either cell cycle dependent or independent ways argues for some level of specificity."***

   These are all germane points raised by the reviewer. We agree that it is likely that SWI/SNF plays a determining role in many if not most other dynamic cell biological processes in the nematode, and we agree that doing an *ama-1* RNAi experiment (or even better an AC-specific depletion of *ama-1::AID*) is worth doing but we hope that as the reviewer suggests, that perhaps this experiment would best be saved for a new paper.

   Having looked at endogenously labeled strains for representative subunits of the core and both assemblies, SWI/SNF appears to be expressed ubiquitously. In the case of the Q neuroblasts, the Oliver Hobert group has looked at mutants in *ham-3/swsn-2.1* and determined no strong role for loss of the SWI/SNF subunit in postembryonic Q cell migration (1). Toward the reviewer's point, in the same study, it is found that loss of *ham-3* does effect CAN cell migration (1). SWI/SNF research in *C. elegans* suffers from lack of a comprehensive literature review of all the reported effects of loss of individual subunits among the ~30 publications on the topic to date. Our group is interested in producing such a review, which we believe will complement this manuscript well. To address these points directly, we have reworked the conclusion of this paper (changes tracked). We

hope the reviewer agrees that the updated discussion section concisely and appropriately considers these points.

2. ***"Another key unanswered question is what molecular defects (presumably in anchor cell gene expression) underly the observed cell cycle and invasion phenotypes when each complex is perturbed. This is eminently addressable with modern techniques but in my view given the already large scope of this study is fine to leave for a future paper."***

   With respect to BAF/*swsn-8* subunit knockdown, DHB measurements suggest that a significant fraction or perhaps most of the AC invasion phenotype is an indirect consequence of loss of G0 arrest. Previous work in our lab has determined that the AC must arrest in G0 to invade the BM and to date, in terms of the cell cycle regulatory machinery, we have only identified the cyclin-dependent kinase inhibitor *cki-1* as a regulator of cell cycle arrest in the AC (manuscript ref 23, Matus et al. 2015). We have shown previously that inappropriate cell cycle entrance results in loss of MMP expression (*zmp-1, -3* and *-6)* and downregulation of transcriptional reporters for F-actin regulators such as *unc-34*/Ena-Vas and *exc-6*/Formin (manuscript ref. 23, Matus et al 2015). We are currently preparing a manuscript trying to better understand the connection between cell cycle regulation and invasion, but that is beyond the scope of the current work.

   For PBAF loss, we demonstrate that at least part of the defect in AC invasion is a consequence of dysregulation of the *fos-1*/FOS pro-invasive TF. Fos has been previously studied in the context of ECM remodeling and BM attachment together with Netrin signaling. In this paper, we attempted to phenotypically characterize the different invasion defects given loss of individual subunits. Our lab is currently working on a follow up paper, which explores the specific targets of the BAF and PBAF assemblies.

Minor comments:

1. ***I would move figure S2/S3 to be a main figure***

   We thank the reviewer for the suggestion and have combined and moved Figures S2/3

2. ***Figure 7G – are all the 'NS' bars necessary? Would be easier to follow if you just state in the legend that other comparisons are NS, highlighting the few significant comparisons*.**

   We see the reviewer's point and have removed all NS bars in this panel

3. ***Figure callouts for Figure 7 in the text are not right (for example Figure 7E for mNG quantification, 7F for "Detached" phenotype example***

   We have updated and corrected all figure callouts to reflect the error pointed out by the reviewer in this section and the recommendation of moving supplemental figures 2/3 to the main body of the manuscript

"Reviewer #3: *This manuscript by Smith et al uses the Anchor cell invasion in C elegans as a model system to identify new regulators of invasion. Using this elegant model system and genetic screens they find:*

• SWI/SNF and its sub-complexes pBAF and BAF to control AC cell invasion.
• SWI/SNF acts in a dose dependent manner to regulate AC cell invasion.
• The PBAF and BAF control different aspects of invasion. While PBAF controls invasion via Fos and attachment to the basement membrane, BAF controls invasion via the cell cycle.

This manuscript has already undergone one round of review process and the authors have satisfactorily addressed the points brought up by the previous round of review. I agree with the authors that direct targets of these complexes are difficult to obtain currently due to limitations in current technology. In addition, I also feel that this manuscript has sufficient data to contribute significantly to the field."

Major comments: ***"****My major issue is how the manuscript is written. As an outsider to the C elegans field, and contrary to the previous round of reviews, I thought it was a difficult manuscript read and navigate. Here are a few suggestions that the authors can use to simplify the manuscript so it can be accessible to the broader scientific community if they wish."*

We greatly appreciate this feedback! It is our intention to make the manuscript as accessible as possible to all readers, including those who don't use *C. elegans* as a research organism. Most of the time we have spent in editing this manuscript since receiving the *PLoS Genetics* reviews has been in addressing this point. We hope the reviewer will agree that the updated version is more accessible, having avoided CRFs and other unnecessary acronyms.

1. ***Use fewer acronyms. I feel like acronyms like "CRFs" are not required and one can spell this out. There are a lot of moving parts to this manuscript—using fewer acronyms will allow the reader to focus on the science.***

   We have taken the reviewer's point seriously and removed all CRF acronyms in the paper. We have also taken time to review all other acronyms and short hands in the paper for the purpose of increasing broader accessibility.

2. ***While reading the initial part of the manuscript I was deeply worried about RNAi levels and phenotypic analysis. This was addressed in the latter part of the manuscript by using nulls – can they indicate to readers that this is in the offing? In my opinion, it will be easier to combine these sections and reorganize, but I also realize this is a lot of work. Any effort to make the manuscript more accessible will be good.***

   As the reviewer suggests, reliance on RNAi alone could be problematic, due to the incomplete penetrance / variation between tissues and normally an inability to accurately assess loss. We would argue that in our manuscript, we attempted to combat these potential limitations using RNAi by quantifying RNAi efficacy by measuring loss of endogenously tagged SWI/SNF subunits when treated with RNAi. In support of this, **Reviewer 2** commented:

   "The authors should be commended for using their GFP CRISPR lines to measure the extent of RNAi-mediated knockdown, something that is unfortunately rarely done in *C. elegans* studies."

   Additionally, the first thing we did after the initial RNAi screen was confirm AC defects using two established temperature sensitive hypomorphic alleles (line 189). Next, we establish the efficacy of improved RNAi vectors (line 204) and then pair these improved RNAi vectors with endogenously GFP-tagged strains (line 253). Finally, we utilize a GFP-nanobody-degradation approach to deplete the core ATPase away, pairing this with RNAi to generate as close to a "null" phenotype as we can in our system.

3. ***In general it is not a good idea to point to things that are not there in figures. For example, in Figure 3E-D there are arrows and lines showing cells that are not there. I would draw outlines in this case or at least show the merge first. Again, this would make it accessible to a broader audience who are not familiar with AC cell invasion model and what they should look for.***

   We have addressed this point and updated Figure 3 to reflect the reviewer's comment, agreeing that it is better not to draw attention to missing cells/empty space. Instead, we have traced/outlined cells in panels where we are drawing the reader's attention to lack or loss of expression, for example. Pink or black dotted lines have been added to figures to indicate anchor cell boundaries and arrowheads are used to indicate lack of expression in AC nuclei.

4. ***It has taken a lot of work for the authors to show that PBAF regulates FOS and BAF cell cycle. Why do they use "?" in the model figure? Maybe use a***

dotted line? Or may say “other factors?”

We are sensitive to the limitations of our analysis, namely that we have not identified the specific targets of the BAF and PBAF accessory complexes. Question marks were included to reflect this. However, it is important to us to avoid suggesting a lack of confidence in our conclusions; the reviewer’s point is well taken. We have removed the question marks and believe that cautious language in addressing the summary figure should more clearly express remaining questions posed by the model.

5. ***The discussion is long and can be tightened a bit—this does not mean that they need to remove speculative sections.***

   Per this reviewer comments, along with those of other reviewers, we have made the discussion more concise, reducing the word length by approximately ~10% in the revised submission.
