# Supplementary material for: The SWI/SNF chromatin remodeling assemblies BAF and PBAF differentially regulate cell cycle exit and cellular invasion in vivo
Source: PLoS Genet. 2022 Jan 4;18(1):e1009981. doi: 10.1371/journal.pgen.1009981 (PMC8759636; doi:10.1371/journal.pgen.1009981)
Supplement: Attachment — Submitted filename: decion_letter_plos_biology.docx [file pgen.1009981.s013.docx]

Dear Dr Matus,

Thank you very much for submitting your manuscript "The SWI/SNF chromatin remodeling assemblies BAF and PBAF differentially regulate cell cycle exit and cellular invasion in vivo" as a Research Article for review by PLOS Biology. Please accept my apologies for the delay in the processing of your manuscript during peer review. As with all papers reviewed by the journal, yours was assessed and discussed by the PLOS Biology editors, by an academic editor with relevant expertise, and by three independent reviewers. Based on the reviews, I regret that we will not be pursuing this manuscript for publication in the journal. I hope that you will find the reviewers' feedback helpful as you consider how to proceed with this work.

The reviews are attached below. As you will see, whilst the reviewers agree that the data is of high quality, they also raise overlapping concerns with the level of mechanistic insight in the manuscript. Specifically, they note that the data does not directly identify targets of BAF/PBAF that regulate anchor cell invasion and that it relies on the modulation of transcription factors which can have indirect effects. Considering all the concerns and after a discussion with the academic editor, I am sorry to say that we have concluded that the level of mechanistic insight in the manuscript does not provide the strength of advance that we are striving to achieve at the journal.

The reviews are attached, and we hope they may help you should you decide to revise the manuscript for submission elsewhere. I am sorry that we cannot be more positive on this occasion.

I hope you appreciate the reasons for this decision and will consider PLOS Biology for other submissions in the future. Thank you for your support of PLOS and of Open Access publishing.

Sincerely,

Richard

Richard Hodge, PhD
Associate Editor, PLOS Biology
rhodge@plos.org

------------------------------------------------------------------------

Reviewer remarks:


Reviewer #1: This manuscript provides novel insights into the role of different C. elegans SWI/SNF chromatin remodeling complexes in the regulation of cellular invasion. Recent studies have shown that mutations in many subunits of this complex can lead to developmental disorders as well as promotion of tumor development and progression. However, the pathogenic mechanisms by which loss of different SWI/SNF complexes contribute to development of these diseases remains unresolved. The authors have taken advantage of a defined normal developmental process in C. elegans that involves cellular invasion to dissect the contribution of 2 major SWI/SNF complexes, BAF and PBAF, in invasion. Their results implicate the BAF complex as the regulator of cellular proliferation in this process and the PBAF complex in the control of cell-cell contact and subsequent basement membrane remodeling. The authors present clear and convincing data to support their notion of the differing roles of these complexes in cellular invasion. However, the authors did not consider several important issues regarding the composition and levels of the different SWI/SNF complexes in their studies. In addition, the authors should discuss how the limitations of these results in this C. elegans model in understanding the regulation of tumor invasion in humans.

Major Comments:

1. Why did the swsn4 ATPase subunit not show up as a hit in Table S2, especially because the authors use it for most of the studies?

2) Does C. elegans not contain GBAF complexes? If this organism possesses this third major type of SWI/SNF complexes, the authors why did not consider this complex in their studies.

3) Differential stability of SWI/SNF complex members has been shown in human cells. Considering that the authors may also observed differential stability of SWI/SNF complex members in their studies (Fig. S4), have they considered how loss of different subunits may affect expression of other subunits, especially in the context of BAF vs. PBAF activities?

4) The authors should discuss whether the different levels of depletion of swsn4 equally reduces the assembly of the BAF and PBAF complexes. In other words, does a 90% reduction of the swsn4 protein result in concomitant 90% reductions in the number of BAF and PBAF complexes?

5) The authors emphasize the broad range of C/N ratios observed in the swsn4 knockdowns shown in Figure 4F. However, it appears that the same broad range appears in the swsn8 knockdowns in the same panel. Could the authors provide a further explanation for their interpretation of these data?

6) Did the authors ever observe complete penetration through the basement membrane in the swsn8 knockdowns upon CKI-1 expression? The results shown in Figures 5 and S6 show incomplete penetrance of the BM.

7) Multiple reports in human cells have shown that loss of SWI/SNF complex activity results in altered localization of the FOS family of TFs without significant changes in FOS protein levels (1-3). The authors should discuss this potential contribution altered fos-1 binding, even in the absence of reduced protein levels, to their studies.

8) The authors should consider in their discussion that their data does not measure the effects on the composition and/or levels of SWI/SNF complexes after knockdown of the different subunits. The unstated assumption throughout the study is that decreased expression of SWSN4, SWSN8 and PBRM1 proteins reduces the levels of intact BAF and/or PBAF complexes in a proportional manner. However, other reports have shown that loss of ARID1A expression affects the levels and localization of PBAF complexes in human cells (4, 5).

9) The sections in the Discussion entitled "The SWI/SNF BAF assembly promotes cellular invasion through induction of G0 cell cycle arrest" and "The PBAF assembly regulates AC invasion and attachment to the BM" are essentially recapitulation of the Results section and should be significantly reduced to cover insights from the results.

Minor Comments:

1) The authors should add p values for Figure S5 panels B and C.

2) The authors should discuss references, such as Orvis et al. and Wang et al., that demonstrate that knockdown or knockout of SWI/SNF subunits in human and mouse models of cancer significantly increases invasion of tumors (5, 6).

1. Nakayama RT, Pulice JL, Valencia AM, McBride MJ, McKenzie ZM, Gillespie MA, Ku WL, Teng M, Cui K, Williams RT, Cassel SH, Qing H, Widmer CJ, Demetri GD, Irizarry RA, Zhao K, Ranish JA, Kadoch C. SMARCB1 is required for widespread BAF complex-mediated activation of enhancers and bivalent promoters. Nature Genetics. 2017. doi: 10.1038/ng.3958. PubMed PMID: 28945250.
2. Orlando KA, Douglas AK, Abudu A, Wang Y, Tessier-Cloutier B, Su W, Peters A, Sherman LS, Moore R, Nguyen V, Negri GL, Colborne S, Morin GB, Kommoss F, Lang JD, Hendricks WP, Raupach EA, Pirrotte P, Huntsman DG, Trent JM, Parker JS, Raab JR, Weissman BE. Re-expression of SMARCA4/BRG1 in small cell carcinoma of ovary, hypercalcemic type (SCCOHT) promotes an epithelial-like gene signature through an AP-1-dependent mechanism. Elife. 2020;9. Epub 2020/12/24. doi: 10.7554/eLife.59073. PubMed PMID: 33355532.
3. Wang X, Lee RS, Alver BH, Haswell JR, Wang S, Mieczkowski J, Drier Y, Gillespie SM, Archer TC, Wu JN, Tzvetkov EP, Troisi EC, Pomeroy SL, Biegel JA, Tolstorukov MY, Bernstein BE, Park PJ, Roberts CW. SMARCB1-mediated SWI/SNF complex function is essential for enhancer regulation. Nat Genet. 2017;49(2):289-95. doi: 10.1038/ng.3746. PubMed PMID: 27941797; PMCID: PMC5285474.
4. Raab JR, Resnick S, Magnuson T. Genome-Wide Transcriptional Regulation Mediated by Biochemically Distinct SWI/SNF Complexes. PLoS Genetics. 2015;11(12):e1005748. doi: 10.1371/journal.pgen.1005748. PubMed PMID: 26716708; PMCID: PMC4699898.
5. Wang Z, Chen K, Jia Y, Chuang J-C, Sun X, Lin Y-H, Celen C, Li L, Huang F, Liu X, Castrillon DH, Wang T, Zhu H. Dual ARID1A/ARID1B loss leads to rapid carcinogenesis and disruptive redistribution of BAF complexes. Nature Cancer. 2020;1(9):909-22. doi: 10.1038/s43018-020-00109-0.
6. Orvis T, Hepperla A, Walter V, Song S, Simon J, Parker J, Wilkerson MD, Desai N, Major MB, Hayes DN, Davis IJ, Weissman B. BRG1/SMARCA4 inactivation promotes non-small cell lung cancer aggressiveness by altering chromatin organization. Cancer research. 2014;74(22):6486-98. doi: 10.1158/0008-5472.CAN-14-0061. PubMed PMID: 25115300; PMCID: PMC4233181.


Reviewer #2: Smith et al. have used the AC invasion model in C. elegans to study the role of chromatin regulators (CRF) during cell invasion. A systematic RNAi screen identified a large number of CRFs -as the authors already expected- but also highlighted the importance of the SWI/SNF complex for cell invasion. In particular, the authors show that the two distinct SWI/SNF complexes, BAF and PBAF, play distinct roles during invasion. While the BAF complex acts predominantly as a cell cycle repressor to prevent the AC from dividing and thereby permit invasion, the PBAF complex seems to act independently of the cell cycle by promoting basement membrane attachment of the AC and acting together with the fos-1 proto-oncogene homolog.
The manuscript consists of an elegant in-depth phenotypic analysis of the invasion defects observed after down-regulating different SWI/SNF components to different degrees. The experiments are well-executed and use state of the art techniques to interfere with SWI/SNF function, such as tissue-specific GFP nanonbody expression and the auxin-inducible protein degradation system. Overall, the manuscript is very well written. The discussion is somewhat broad - though very interesting as it contains a review-style overview of the many SWI/SNF functions- but I'd prefer a more to the point, critical discussion of the actual data mentioning alternative explanations of the results (see specific comments below).
The obvious limitation of this work is the absence of a molecular mechanism, i.e. the identification of critical invasion regulators that are direct targets of the BAF and PBAF complexes. Since invasion is controlled by a complex network of transcription factors (TFs) with multiple feedback loops, altering expression of one TF will affect many others. Hence, it is very difficult to distinguish direct from indirect effects by observing TF reporter expression after SWI/SNF knock-down or knock-out. Overall, this study is of high quality. My only concern is the absence of a molecular mechanism that would provide conceptually new insight in the role of CREs in cell invasion. I therefore believe it should be an editorial decision whether this manuscript is suitable for publication in PLOS Biology.

Specific comments
1. Many of the experiments rely on quantifying fluorescence signal intensities in the AC. As far as I understand the methods section, the AC body was selected manually to measure the mean fluorescence. I do trust the authors that a 30% reduction can be reliably quantified by this approach, but I think smaller changes (e.g. a 5% reduction measured using a antiGFP nanobody) may be within the error margin of the method. Also, indicating the % reduction (e.g. 38.69% depletion) and penetrance of invasion defects (e.g. 48.39% invasion defect for n=31) with one or two decimal precision overstates the precision. Most if not all % values should therefore be rounded to full values.
2. p.12 describes elevated expression levels of SWI/SNF reporters in the Ac. Is there a cell-cycle dependence of SWI/SNF expression levels, i.e. in dividing vs arrested ACs? Is there a biological significance to the elevated levels of some SWI/SNF components?
3. p.14. The nanobodies to downregulate swsn-4. I do not understand the statement: "In contrast, the egl-43>antiGFP nanobody expressed highly in the AC….(Fig 3C asterisk)" Fig. 3 only shows swsn-4 reporter expression levels , but the antiGFP nanobody expression pattern is not shown. What is the evidence for the tissue-specificity for the antiGFP nanonbody transgenes?
4. Regarding the dosage dependent effects of SWI/SNF: While work in the M lineage has shown that knock-down versus knock-out of swsn-4 genes causes qualitative phenotypic differences, i.e. hyper-proliferation vs proliferation arrest, the data in the AC shown in Fig. 3 point to quantitative differences. Swsn-4 knock-down by RNAi or nanobody causes a weak over-proliferation and a penetrant single AC invasion phenotype, while knock-out (RNAi plus nanobody) a completely penetrant invasion defect with a majority of animals having extra ACs. In my view, this is a fundamental difference from the data in the M lineage.
5. p.16, l9…"arrested in S phase"
6. p.18ff & Fig. 4F describing quantification of the CDK sensor. I do not follow the reasoning allowing the authors to distinguish between G1 ad G0 arrest. The argument is that the AC has a similar ratio as the post-mitotic (G0) Pi cells. However, an essential control would be to compare the AC values to the ratios detected in pre-mitotic G1-arrested cells, e.g. in the seam cells or early VPCs before vulval induction.
7. p.24 6 Fig showing the AID degradation of PBRM-1. Analogous to Fig4 with swsn-4, one should perform pbrm-1 RNAi in the AID strain to inhibit PBRM-1 expression as much as possible- and then assay for AC proliferation.
8. p.26 & Fig. S7: The conclusion of combining the hypomorphic pbrm-1::aid allele with fos-1 RNAi is that the two genes function together to regulate AC invasion independently of the cell cycle. However, this argument is based on combining two partial reduction of function conditions, which is equally consistent with the two genes acting independently in two parallel pathways.


Reviewer #3: Review of Smith et al.

The authors previously showed networks of transcription factors (TFs) regulating either cell cycle arrest and the expression of proinvasive genes, both of which are required for invasion of the anchor cell in C. elegans. In the current manuscript, the authors found that two distinct but related chromatin remodeling complexes, BAF and PBAF are differentially required for these two processes. Although the authors wrote as if these complexes directly regulate the processes (e.g., in Abstract), it can be easily expected that such effects are through the expression of TFs, which, in fact, is affected at least by knock down of ATPase subunit SWSN-4, a common components of BAF and PBAF. In addition, the manuscript failed to thoroughly explore whether their expression is differentially regulated by BAF and PBAF, which can be expected based on the phenotypes caused by their specific inhibition. Therefore, how these chromatin remodelers regulate cell invasion is not clear. I suggest the authors to first describe the functions of these complexes in the regulation of the TFs (more direct effects), and then clearly describe whether the various phenotypes (invasion, cell cycle and attachment to BM) can be explained simply by the effects on TFs or not (possibility of additional functions). In any case, since these complexes are known to regulate the expression of various genes and cell cycle, the findings in this manuscript are no surprise. Although the authors emphasize the conservation of the cell invasion mechanism, I do not think that the transcription factors used in C. elegans invasion have similar functions in other organisms. Therefore, I am not sure how much the functions of the chromatin remodelers in C. elegans are relevant to cell invasion in mammalian cells.

The authors did various experiments to know the null phenotype of the complexes. However, I wonder why they did not use (or make) null mutants. Although the null mutants of the core components are likely to be lethal, those of BAF or PBAF specific components might be used for the analyses. In fact, there are some deletion mutants available from CGC. They may not be null mutants, but making null mutants by CRISPR may be easier than to make degron lines. The current manuscript is too descriptive and complicated about RNAi and degron methodologies. If null mutants can be used, the manuscript is likely to be much simpler.

I wonder how the invasion defects were evaluated. In Fig. 5C', a weak GFP signal of BM apparently remains, although it is described as rescue of invasion, while in Fig. 7B, a similar weak signal is described as partial bleach. (Yellow arrows are on many figures, but I do not find their description in figure legends)

AC detachment was observed by the combination of pbrm-1 inhibition through the degron method and swsn-7(RNAi). Therefore, the fos-1 expression should be analyzed in the same condition rather than pbrm-1(RNAi) in which AC detachment was not observed (Fig S7A).

Page 26 We found that even without the addition of auxin, co-depletion with fos- 1(RNAi)—
PBRM-1::AID is not depleted without auxin.

Together, these results suggest that the PBAF assembly functions with FOS-1 to regulate AC invasion.
I believe that PBAF is upstream of FOS-1. Do the authors think PBAF has an additional role than the regulation of fos-1 expression?
